# Factors influencing the uptake of a mono-PrEP implant for the prevention of HIV: Males’ perspectives from three South African provinces

**DOI:** 10.1371/journal.pone.0296341

**Published:** 2024-01-02

**Authors:** Nqaba Mthimkhulu, Glory Chidumwa, Alison Kutywayo, Paballo Mataboge, Catherine E. Martin, Khanyiswa Kwatsha, Nthabiseng Makalela, Mbali Mazibuko, Vusile Butler, Saiqa Mullick

**Affiliations:** Wits RHI, University of the Witwatersrand, Johannesburg, South Africa; University of Zimbabwe Faculty of Medicine: University of Zimbabwe College of Health Sciences, ZIMBABWE

## Abstract

**Introduction:**

Oral pre-exposure prophylaxis (PrEP) is an effective HIV prevention method; however, males over 15 years face challenges with its effective use. Long-acting prevention products could address barriers to effective PrEP use. This study aimed to estimate the potential uptake of a mono-PrEP implant and the factors influencing uptake among males in South Africa. The study also examined messaging and demand creation tactics that males perceive will improve HIV prevention uptake.

**Methods:**

We conducted a mixed methods study comprising participatory workshops and a self-administered survey among 142 PrEP-eligible males (18–40 years) in three provinces (Gauteng, Eastern Cape, and Kwa-Zulu Natal) in South Africa from July to November 2022. Logistic regression was used to assess the relationship between the potential uptake of a hypothetical, non-biodegradable mono-PrEP implant and socioeconomic and behavioural factors. Workshop data were analysed using content analysis.

**Results:**

The top three HIV prevention products that males would consider using were the monthly pill (74.6%), the mono-PrEP implant (62.7%), and event-driven oral PrEP (59.2%). If one prevention option was available, 31.7% of participants stated that they would use the monthly oral pill, 28.2% would use the six-monthly injection, and 19.7% the mono-PrEP implant. Four key themes were noted as influential to potential mono-PrEP uptake: *“Health Over Everything”*, *“Mono-PrEP Implant Concerns”*, *“Potential Disclosure of Mono-PrEP Use”*, and *“Information Distribution Channels*”. Participants preferred social and mainstream media as information distribution channels to receive information on HIV prevention services, including the mono-PrEP implant.

**Conclusion:**

In this study among predominantly heterosexual men in South Africa, there was interest in long-acting HIV prevention methods but concerns about the mono-PrEP implant. A comprehensive and participatory introduction will be needed for the implant, to improve acceptability and address potential concerns. Demand-creation strategies utilising social media and health campaigns should be considered to engage and reach males.

## Introduction

South Africa bears 19% of the global HIV burden, with an estimated 7.5 million people living with HIV in 2021 [1]. As women are disproportionately affected by HIV compared to men, there is a gap in HIV service provision and coverage of HIV services for men and boys [2]. In 2017, it was estimated that more than 2 million men aged >15 years in South Africa live with HIV, and account for 37% of all adults living with HIV [2]. Heterosexual intercourse and age-disparate relationships among adolescent girls and young women (AGYW) and older men are one of the main methods of HIV transmission [3]. Men (15–59 years) are less likely to test for HIV, with 52.8% of men aged 25–29 years tested for HIV in the last 12 months, compared to 68.4% among females the same age [2]. Men are more likely to have multiple sexual partners compared to women [4,5], engage in high-risk sexual practices [6,7], have inadequate knowledge of HIV and sexually transmitted infections (STIs), and have limited access to appropriate and adequate HIV prevention or care [8,9]. The utilization of HIV preventive programs is further hindered by stigma and discrimination for men, particularly men who have sex with men (MSM) [8,10]. Although heterosexual sex is the primary mode of HIV transmission in Sub-Saharan Africa including South Africa, HIV prevention programs have largely focused on key populations such as AGYW and MSM, often overlooking the needs of heterosexual men [8]. This emphasizes the need for innovative approaches to ensure that men living with or at risk of HIV use services to improve their health outcomes and prevent HIV transmission to their sexual partners [11].

HIV prevention products such as pre-exposure prophylaxis (PrEP) and voluntary medical male circumcision (VMMC) are effective in reducing HIV acquisition [12]. Oral PrEP has been available for men and other individuals at substantial risk of HIV who request PrEP after recognising their vulnerability since 2016 in South Africa [4,13–16]. There is growing PrEP interest and uptake among men in South Africa and previous studies have reported that heterosexual men have a high perception of HIV risk but lack HIV/Sexual reproductive health (SRH) information [17–20]. As of October 2022, 658 885 individuals had been initiated on oral PrEP in South Africa, with data indicating approximately 20% of all PrEP initiations were among men, with those in the 25–34 year age group indicating most interest and uptake [21,22]. PrEP data for males primarily focuses on MSM, while heterosexual males’ data is mainly available in clinical studies in developed and developing countries [17]. A recent South African study revealed that the majority of males initiating PrEP were heterosexual (94.8%, n = 345), with 58% only using PrEP for ≤1 month (early discontinuation) and 18% returning to restart it multiple times [23]. VMMC should be offered as part of a comprehensive HIV prevention package alongside PrEP as it reduces the risk of heterosexual transmission by 60% [24]. Men in South Africa have shown high acceptability and uptake in VMMC; of the 998 213 men who were offered VMMC, over 97% underwent circumcision [24].

Correct use of oral PrEP significantly reduces HIV risk, however, ensuring continued and effective use is still a significant obstacle for end users [4,25,26]. Barriers to effective oral PrEP use include the stigma associated with HIV infection when PrEP is mistaken for antiretroviral therapy (ART), and the fear of disclosing PrEP use to partners [22,26,27]. Other factors including side effects, competing stressors (i.e., unable to prioritize prevention, work schedules physically preventing pill-taking or caretaking roles in the family), low HIV risk perception, and inaccessible health systems have been reported as barriers impacting adherence to PrEP among young men who have sex with men (MSM) and transgender women of colour in America [28]. Similar barriers were reported among heterosexual men in KwaZulu Natal: factors such as PrEP misconceptions, frequent appointments, long waiting times and financial cost to reach the site [18].

Two new methods, injectable cabotegravir (CAB-LA) and a hypothetical mono-PrEP implant, provide long-term protection against HIV [29,30]. These methods present an opportunity to increase PrEP uptake and persistence use among men, particularly heterosexual men, removing barriers to daily pill-taking that prevent effective use [31–34] and providing men with more lifestyle-friendly PrEP options [35]. The mono-PrEP implant, currently a hypothetical product, is conceptualized as a non-biodegradable implant (i.e., removable), which is inserted under the skin of the upper arm, releasing an anti-HIV drug, providing protection for at least one year [36,37]. Potential side effects could include inflammation or itching in the implant area; however, preliminary results from studies evaluating implant safety and tolerability reported that participants found side effects tolerable [36]. Until now, MSM and women have a high level of acceptability for injectable PrEP [32], however, little is known about the attitudes of heterosexual men and their preference for novel long-acting HIV prevention products [35]. It remains unclear how the introduction of a mono-PrEP implant would increase demand for PrEP among males in the country and what their preferred PrEP methods would be. Furthermore, end-users need to be engaged early in product development to ensure that the products developed will be preferred by those they are intended to reach. While the mono-PrEP implant is the focus of this research, there are other HIV prevention methods discussed in this manuscript: once-daily pill (oral PrEP) [13,38], monthly pill, two-monthly injectable, six-monthly injectable and event-driven oral PrEP. The monthly pill is taken once a month, orally to prevent HIV [39]. Event-driven oral PrEP (2 + 1 + 1) involves taking a double dose of PrEP 2–24 hours before expected sex, and if sex happens, an additional pill should be taken 24 hours and 48 hours after the double dose [40]. Long-acting injectable cabotegravir (CAB-LA) is a two-monthly intramuscular injection, given to individuals every eight weeks and has been approved for use in South Africa [41]. The monthly pill and six monthly injectable are currently undergoing clinical trials are not available for public use in South Africa [41,42].

This study aimed to estimate the potential uptake of a non-biodegradable (i.e., removable) PrEP implant (i.e., mono-PrEP implant) among men accessing routine primary healthcare SRH services in South Africa. We also explore educational messaging and demand creation tactics that could improve future uptake of the implant.

## Methods

### Study design

This study presents one component of a larger, mixed method, formative research study conducted by Wits RHI from July to November 2022, which aimed to explore the potential uptake of multi-purpose prevention technology (MPT) implants among women and a mono-PrEP implant among men, as well as healthcare provider considerations for their effective delivery and introduction. This paper reports on the findings from the survey and workshops conducted among male participants.

### Study setting

This study was carried out in three districts in three provinces (i.e., Tshwane District, Gauteng Province; OR Tambo District, Eastern Cape Province; King Cetshwayo District, KwaZulu Natal Province) in nine department of health primary clinics and their linked mobile clinics in South Africa, leveraging the existing geographical footprint of Wits RHI PrEP implementation projects. There is a geographical variation in HIV prevalence in South Africa, with the burden largely concentrated in Gauteng, KwaZulu-Natal, Eastern Cape, Limpopo, and Mpumalanga [43]. Furthermore, the chosen sites have high concentrations of people living with HIV ranging from 12.8% in the Eastern Cape to 26% in Gauteng among women 15–49 years, and ranging from 9.8% in the Eastern Cape to 28.9% in Gauteng among men (15–59 years) [43].

### Study population and recruitment

Males aged 18–40 years accessing SRH services at the facility and mobile sites were recruited, between July and November 2022, by trained fieldworkers, using two recruitment strategies: consecutive sampling and snowballing. Potential participants were approached, screened for eligibility, and provided with an overview of the study. They were eligible for study participation if they were eligible for PrEP (determined by a self-reported HIV-negative status) and were willing and able to consent to participate in the study. If they were interested, they were invited to a workshop session a week later. The snowball sampling strategy involved asking participants to refer eligible, interested friends or family members to participate in the study using a recruitment flyer. The flyer contained the study contact number, allowing interested participants to contact the study team for further information. Potential participants who contacted the study team were screened for eligibility and, if eligible, provided with additional information about the study and invited to the workshop scheduled a few days later.

### Study procedures

The study activities included an information session, and participant workshops with participatory research activities [44], followed by a tablet-based self-administered survey. Participatory action research (PAR) employs a systematic and collaborative approach that involves individuals who are directly affected by issues under study so that they have the opportunity to share their experiences, views, and needs [44]. Throughout the PAR research process, the participant is an expert and they are actively involved and have the opportunity to express their opinions and they are considered [44,45]. This study adopted the PAR approach to examine the educational messaging and demand creation tactics proposed by participants to improve the uptake of HIV prevention methods, including a mono-PrEP implant.

All participants participated in an information session and completed a self-administered survey, and a subset participated in the PAR workshops. The recruitment for the workshops was carried out until data saturation was reached. Four workshops were conducted: one in King Cetshwayo District, one in OR Tambo District, and two in Tshwane between July and August 2022. On average, there were 13 participants per workshop, and the workshops were held in a local hotel or community venue and facilitated by the trained study team. First, participants were provided with an information session of approximately 45 minutes, presented by a trained study member, on existing and near-market HIV prevention methods, including the hypothetical one-year multipurpose prevention technology (MPT) implant (i.e., preventing pregnancy and HIV simultaneously for females) and the hypothetical one-year mono-PrEP implant (i.e., prevents HIV only) for males. The presentation covered the study’s aims and objectives and the following topics: product description, potential side effects, duration of prevention, product administration, effectiveness, and anticipated availability. Participant questions were answered throughout.

**Fig 1:**
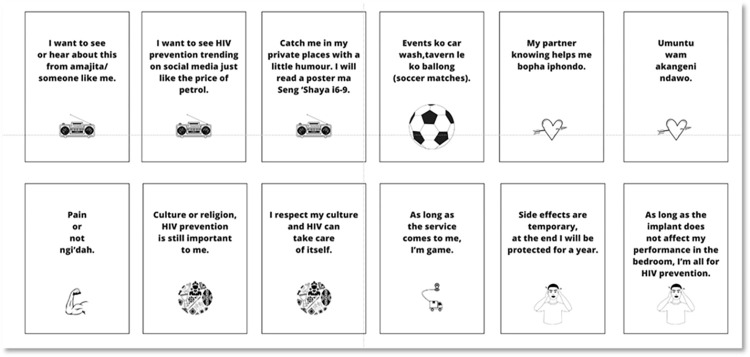
Men’s conference activity cards.

Following this, participants were invited to take part in the PAR activity: a group card sorting activity [46] called *“Men’s conference”*. The aim was to elicit participants’ views on the mono-PrEP implant and preferred educational messages and demand creation tactics which may improve product uptake. The facilitator shared 12 cards (Fig 1) with each group of men, reflecting potential messages that could be adopted to encourage men to use prevention products. As a group, participants were asked to select six of the 12 cards that they deemed important to influencing their decision-making process to use the mono-PrEP implant. A discussion then ensued, with the facilitator probing, allowing participants to elaborate on their groups’ card choices. Each workshop was observed by at least two trained study team members with observations documented using a standard observation guide. The observation guide aimed to capture the discussion points, participant questions, and reflections. Observers were partial participants [47] and took part in the interactions in the workshop but not in the specific PAR workshop activity. Following the PAR workshops, participants completed a self-administered survey on a handheld tablet, cell phone, or computer. The survey was anonymous and collected information on participant demographics, sexual behaviour and risk perceptions, and experiences of HIV prevention and condom use. A section on HIV prevention product preferences and acceptability, including the mono-PrEP implant, and the decision-making factors involved in choosing a prevention method were also included.

Once the PAR workshop sample was reached, subsequent participants were recruited to only attend the information session and complete the survey. Participants were given the same 45-minute information session on new and existing HIV prevention strategies, including MPT and mono-PrEP implants, before completing the same self-administered survey. Research activities were conducted predominantly in English however when participants were unclear about certain phrases or terminology during the workshop, the facilitator would clarify using the local language.

### Data management and analysis

#### Qualitative data analysis (Workshops)

Observation notes from each workshop were transcribed in English by each observer and consolidated by the Researcher (PM) and the Associate Researcher (NM) to produce one consolidated observation note from each workshop for analysis. The consolidated observation notes were uploaded to a central location where access was restricted to the study team before the consolidated notes were imported and coded in QSR NVIVO 12 [48]. The data was coded by the Associate Researcher (NM) with assistance from the Researcher (PM) to ensure intercoder reliability. The Associate Researcher (NM) then refined the code list and coded all the transcripts; the parent codes were identified deductively using the observation guides, while the sub-codes were identified inductively based on the data. Ten codes were created from the dataset: namely *‘Access to accurate information’*, *‘Access to healthcare services’*, *‘Concerns about prevention product’*, *‘Information channels for sharing PrEP Information’*, *‘Providers of Information’*, *‘Other prevention products’*, *‘Risky behaviours’*, *‘Stigma’*, *‘Importance of disclosing use of prevention products’*, *‘Factors influencing use’*. The codes were further grouped into four major themes namely (1) **Health over everything,** (2) **Mono-PrEP implant concerns**, (3) **Disclosing Mono-PrEP use**, and (4) **Information distribution channels**. Content analysis was used to identify, describe, and explore patterns and common themes.

#### Quantitative data analysis (survey)

The survey was developed and managed by the study team using Research Electronic Data Capture (REDCap) hosted at the University of the Witwatersrand [49,50]. The survey was self-administered and in English; if a participant needed help, a member of the study team assisted the participant in completing the survey. Self-perceived risk of HIV and STIs was reported as either “no perceived risk” or “some perceived risk”, for those who reported being “a little”, “somewhat”, or “very much” at risk based on their risk perception. Early sexual debut was defined as having first sexual intercourse at the age of 14 years or younger. Transactional sex was defined as ever having had a sexual relationship with benefits such as money, food, or a place to stay. The variable age was a continuous variable and was categorized as "18–24 years" (1) and "25–40 years" (2).

Data on the factors influencing HIV prevention method choice were collected. Participants were presented with 15 factors, based on a previous MPT acceptability study in Uganda, Nigeria, and South Africa [51], identified to influence decision-making when choosing a prevention product. These were: (1) Side effects are manageable; (2) Size of product; (3) Convenient to use; (4) Flexible for my lifestyle/suits my lifestyle; (5) Available for use; (6) Avoidance of painful removal and scarring; (7) Effectiveness; (8) Frequency of dosing; (9) Long term protection; (10) Administration by a healthcare provider; (11) Low user burden; (12) Discreet/privacy; (13) Method of use (such as pill, insertion into vagina or arm, injection); (14) Provides dual protection; (15) Dissolvable. Participants were asked to rank these in order of importance from 1 to 15. The most important factor in the choice of an HIV prevention method was defined as the factor ranked number one (most important) by each participant.

Participants were presented with different prevention methods and were asked, for each method, if they would consider using it to protect themselves from getting HIV. The options included: (1) once daily pill; (2) monthly pill; (3) two-monthly injectable; (4) six-monthly injectable; (5) event-driven oral PrEP and (6) mono-PrEP implant. Participants were then asked to select the prevention product they would choose if only one method was available to them. Based on their responses, participants were categorised as either preferring ***“mono-PrEP implant”*** if they reported that they would consider using a mono-PrEP implant; or preferring ***“Other PrEP methods”*** if they reported that they would consider other PrEP methods (i.e., once-daily pill; monthly pill; two-monthly injectable; six-monthly injectable; event-driven oral pill). Those who indicated they would use none of the products were categorized as not choosing any form of PrEP.

### Statistical analysis

All analyses were conducted with STATA v.15 [52]. Descriptive statistics were conducted to describe the study population characteristics (i.e., socio-demographic factors, sexual behaviour, and perceived risk of HIV and STIs). Factors that were deemed important to males when choosing a prevention product, what males wanted to prevent, and their preferred HIV prevention method were explored descriptively. We conducted a logistic regression analysis, adjusting for age and workshop participation, to determine the factors associated with choosing a mono-PrEP implant as their preferred prevention option (if only one option was available). Statistical significance was set at 5%.

### Ethical approvals and considerations

Ethics approval for the study was granted by the Human Research Ethics Committee of the University of the Witwatersrand (M220305). Provincial research approval was also granted. Written informed consent was sought from participants for study participation. All participants were reimbursed R50 ($2.74) for transport. Participants who participated in the information session, and PAR workshop and completed the survey received R200 ($10.95). Participants who attended the information session and completed the survey only received ZAR100 ($5.78).

## Results

After screening for eligibility, 184 participants were eligible, and 145 (79%) were enrolled in the study. Participants were considered enrolled in the study if they attended the workshop or completed a survey. Due to time constraints, 3 out of 145 (2%) participants attended the workshop but did not complete the survey, therefore the final analysis sample included 142 men who completed the survey. Out of the 142 males enrolled, 55 (39%) participated in the workshop and 87 (61%) participated in the information session only. Of the 142 males enrolled, 70 (49%) were recruited through the facility, 66 (46%) were recruited through snowballing and 6 (4%) were recruited at the mobile van.

### Socio-demographic and sexual behavioural factors

The demographic characteristics of participants are presented in **Table 1,** stratified by age. Of the 142 participants in our analysis, over 60% (n = 95) were 25–40 years and 46.5% (n = 66) were from Gauteng province. Most of the participants (93.0%, n = 132), indicated they were heterosexual, 47.2% (n = 67) had completed college or university and over half were unemployed (54.2%, n = 77) at the time of the survey.

**Table 1:** Socio-demographic and sexual behavioral factors of survey participants by population group.

	Males 18–24 years N = 47 (33%)	Males 25–40 years N = 95 (70%)	Total N = 142 (100%)
	N (%)	N (%)	N (%)
**Demographic Characteristics**			
***Study Site***			
KZN	12 (25.5%)	23 (24.2%)	35 (24.6%)
Gauteng	23 (48.9%)	43 (45.3%)	66 (46.5%)
Eastern Cape	12 (25.5%)	29 (30.5%)	41 (28.9%)
***Sexual Orientation***			
Heterosexual	45 (95.7%)	87 (91.6%)	132 (93.0%)
Bisexual/Homosexual	2 (4.3%)	8 (8.4%)	10 (7.0%)
***Highest level of education completed***			
Primary school or lower	1 (2.1%)	4 (4.2%)	5 (3.5%)
Secondary school	29 (61.7%)	37 (38.9%)	66 (46.5%)
College or University	16 (34.0%)	51 (53.7%)	67 (47.2%)
Unknown	1 (2.1%)	3 (3.2%)	4 (2.8%)
***Employment status***			
Student	12 (25.5%)	5 (5.3%)	17 (12.0%)
Employed	6 (12.8%)	40 (42.1%)	46 (32.4%)
Unemployed	28 (59.6%)	49 (51.6%)	77 (54.2%)
Unknown	1 (2.1%)	1 (1.1%)	2 (1.4%)
**Sexual behaviour**			
***Ever had sexual intercourse***	36 (76.6%)	83 (87.4%)	119 (83.8%)
***Early sexual debut***	7 (14.9%)	19 (20.0%)	26 (18.3%)
***Relationship status***			
Single (no sexual partner)^1^	12 (25.5%)	23 (24.2%)	35 (24.6%)
Casual partners	22 (46.8%)	34 (35.8%)	56 (39.4%)
Married or committed relationship^2^	11 (23.4%)	32 (33.7%)	43 (30.3%)
Other/Unknown	2 (4.3%)	6 (6.3%)	8 (5.6%)
***Knowledge of primary partner’s HIV status*** ^***3***^			
Unknown	19 (54.29%)	24 (33.33%)	43 (40.19)
Known	16 (45.71%)	48 (66.67%)	64 (59.81)
***More than one sexual partner***	15 (42.86%)	35 (48.61%)	50 (46.73%)
***Condom use at last sex***	24 (51.1%)	50 (52.6%)	74 (52.1%)
***Ever had transactional sex***	14 (29.8%)	29 (30.5%)	43 (30.3%)
***Ever tested for HIV***	43 (91.5%)	90 (94.7%)	133 (93.7%)
***Ever had an STI***	12 (25.5%)	33 (34.7%)	45 (31.7%)
***Some perceived risk of HIV***	8 (17.0%)	31 (32.6%)	39 (27.5%)
***Some perceived risk of STI***	16 (34.0%)	38 (40.0%)	54 (38.0%)
***Ever used PrEP***	8 (17.0%)	26 (27.4%)	34 (23.9%)
***Ever used PEP***	6 (12.8%)	18 (18.9%)	24 (16.9%)

^1^ Single refers to participants who are single and have no sexual partner.

^2^ Refers to individuals who are not married and are either cohabitating or in a long-term relationship with their primary partner.

^3^ Of those who had a partner (n = 107).

More than 80% (83.8%, n = 119) of participants reported ever having sexual intercourse, with early sexual debut ranging from 14.9% (n = 7) among males 18–24 years to 20% (n = 26) among older males. Over a third (39.4% n = 56) of the participants reported being in causal relationships. Over half of the males (59.8%, n = 64) reported that they knew their primary partner’s HIV status. Among males >25 years, 48.61% (n = 35) reported having more than one sexual partner and 52.1% (n = 50) reported using a condom at last sex. Over 90% of males reported that they had ever tested for HIV (n = 133) and 31.7% (n = 45) of males reported that they had ever had an STI. Just under three-quarters (72.5%, n = 103) of the men did not perceive themselves as at risk of HIV (72.5%, n = 103) or an STI (62.0%, n = 88). Of the 142 participants, 27.4% of males >25 years (n = 26) and 17.0% (n = 8) of males <24 years stated that they had used PrEP. Seventeen percent of males reported ever using post-exposure prophylaxis (PEP) (16.9%, n = 24).

### Potential uptake of a mono-PrEP implant

Most participants (88.7%, n = 126) wanted to prevent both HIV and STIs (Table 2). The factors identified as being the most important when choosing a prevention product were whether the side effects are manageable (29.6%, n = 42), followed by whether the product offers long-term protection (19.0%, n = 27), and whether the product offers dual protection (HIV/STI) (12.7%, n = 18). The size of the product and method of use were reported to be the most important considerations among 5.6% (n = 8) of males each. Less than 5% of males reported that avoidance of painful removal and scarring, frequency of dosing and requirement for administration by a healthcare provider were important factors when choosing a product.

**Table 2:** Factors influencing men’s choice of prevention products (N = 142).

***What you would like to prevent***	*N = 142*	*%*
STIs only	5	3.5%
HIV only	9	6.3%
HIV and STIs	126	88.7%
Unknown	2	1.4%
***Most important factor in choosing a prevention product***		
Side effects are manageable	42	29.6%
Offers long-term protection	27	19.0%
Provides dual protection	18	12.7%
Size of product	8	5.6%
Method of use (pill/injectable/implant)	8	5.6%
Effectiveness	6	4.2%
Convenient to use	6	4.2%
Dissolvable	5	3.5%
Flexible/suits my lifestyle	4	2.8%
Discreet/Privacy	3	2.1%
Available for use	3	2.1%
Low user burden	3	2.1%
Avoidance of painful removal and scarring	2	1.4%
Frequency of dosing	2	1.4%
Administration by HCP required	1	0.7%
Missing	4	2.8%

The proportion of males willing to consider using various HIV prevention options is presented in Table 3. Product preference varied: from 43.7% (n = 62) who would consider the two monthly injectable, to 74.6% (n = 106) who would consider a monthly pill, with 62.7% (n = 89) willing to consider the mono-PrEP implant. If they could only choose one prevention option, the most frequently chosen product was the monthly pill (31.7%, n = 45), followed by the six-monthly injectable (28.2%, n = 40) and the mono PrEP implant (19.7%, n = 28). The least frequently chosen options were event-driven oral PrEP (2.1%, n = 3) and the two monthly injectable (4.9%. n = 7). Two participants (1.4%) reported that they would not consider using PrEP.

**Table 3:** Proportion of male participants who would consider using various PrEP products (N = 142).

	Participated in workshop and survey (N = 55 (39%))	Participated in information session and survey only (N = 87 (61%))	Total (N = 142 (100%))
	No (%)	No (%)	No (%)
***Would consider using***			
Once daily pill	29 (40.9%)	42 (59.1%)	71 (50.0%)
Monthly pill	36 (34.0%)	70 (66.0%)	106 (74.6%)
Two monthly injectable	21 (33.9%)	41 (66.1%)	62 (43.7%)
Six monthly injectable	24 (70.7%)	58 (29.3%)	82 (57.7%)
Event-driven oral PrEP	30 (35.7%)	54 (64.3%)	84 (59.2%)
Mono-PrEP implant	35 (39.3%)	54 (60.7%)	89 (62.7%)
***Choice if only one prevention method is available***			
Once daily pill	8 (14.6%)	7 (8.1%)	15 (10.6%)
Monthly pill	15 (27.3%)	30 (34.5%)	45 (31.7%)
Two monthly injectable	3 (5.5%)	4 (4.6%)	7 (4.9%)
Six monthly injectable	14 (25.5%)	26 (29.9%)	40 (28.2%)
Event-driven oral PrEP	1 (1.8%)	2 (2.3%)	3 (2.1%)
Mono-PrEP implant	13 (23.6%)	15 (17.2%)	28 (19.7%)
None	1 (1.82%)	1 (1.82%)	2 (1.4%)
Missing	0 (0.0%)	2 (2.3%)	2 (1.4%)

Adjusting for age and workshop attendance, participants residing in Gauteng were more likely to choose a mono-PrEP implant as a prevention product (OR 3.89, 95% CI 1.08–14.69) compared to those residing in KwaZulu Natal. No other factors were associated with the choice of mono-PrEP implant compared to other prevention choices among men (S1 Table).

### Men’s conference

Two-thirds of the participants (62%, n = 88) participated in a PAR workshop. The analysis of the *men’s conference* activity identified four key themes that may influence mono-PrEP uptake: Health over everything, mono-PrEP implant concerns, potential disclosure of mono-PrEP use, HIV prevention product preferences, and information distribution channels.

#### Health over everything

Participants noted that cultural traditions may hinder the use of HIV prevention methods including mono-PrEP implant, as with the use of condoms. Participants had different views on the HIV prevention products (i.e., injections, implants, and oral PrEP) they were presented with. Some stated that they prefer an injection or implant over using oral PrEP because they are likely to forget to take their pills. Some participants stated that their well-being and health have precedence over cultural beliefs and stigma. They acknowledged that tradition, cultural beliefs, and practices are an important element in their daily lives and health, however, health is more important. They further stated that their decision to use an implant would not be influenced by their culture because HIV is a serious problem in South Africa, and they want to protect themselves. Some participants preferred the injection because it is convenient and requires fewer visits to the clinic. In general, participants stated that it does not matter which HIV prevention product they use if they are protected.

#### Mono-PrEP implant concerns

Most participants were concerned about the potential side effects of the mono-PrEP implant as well as the pain during implant insertion and removal. They further stated that the sensation or movement of the implant after insertion would discourage them. They were also concerned about feeling the implant when doing manual labour or lifting heavy items and voiced concerns about the impact on sexual performance including erectile dysfunction and fertility. Participants were concerned about how erectile dysfunction can affect their relationships because their partners would not hesitate to inform their friends. Other participants stated that they can handle pain, especially since the pain is temporary, likening it to the pain to that of circumcision and a soccer injury: they would prefer to be safe than regret it later.

*Disclosure of mono-PrEP implant use*. Some participants felt it was important for their partners to know that they are using an HIV prevention product, to be able to discuss the benefits of the product and to reach a mutual agreement, as trust is important in a relationship. An additional benefit of partner disclosure was that they may encourage them to collect their pills or use the implant, supporting continuation or effective use. However, others felt that disclosing HIV prevention product use to their partner would be problematic, as their partners may assume that they have multiple partners, resulting in a break of trust in the relationship. Some men stated that they are naturally secretive and would not disclose the use of a mono-PrEP implant. For example, they do not inform their partner about the use of *‘imbiza’* (i.e., refers to libido and virility enhancer).

*Information distribution channels*. When reflecting on the appropriate information distribution channels for men, social media and main media were preferred. Participants stated that social media platforms (Twitter, TikTok, WhatsApp, Instagram, and Facebook) could be used to address the stigma surrounding HIV prevention methods and create more awareness. Social media could help people in rural areas easily access HIV prevention information on their phones. Participants indicated that influencers and celebrities should be utilized to share this information and their views on PrEP, as they have a large following. Participants also suggested the use of local soapies (i.e., serialized drama that is broadcast locally on radio or television typically depicting local issues) and adverts to promote the mono-PrEP implant. The sharing of misinformation and fake news was identified as a problem on social media. Overall, participants stated that information on HIV prevention products should be shared moderately because when it is shared frequently, it will annoy them. For example, participants stated that at times insurance adverts are played frequently, leaving them feeling overwhelmed and frustrated.

Additional information distribution channels included places where men usually go, such as car washes, sports fields, and taverns. Men usually meet at these places and discuss their relationships and women, so a discussion on HIV prevention methods would be relevant. However, some participants were against using these platforms for HIV prevention information, stating that other men would judge them. Some felt that when they are at taverns and the sports field, they are there to drink alcohol and watch soccer: they would prefer to receive HIV prevention information on a one-on-one basis rather than in a group. Community health campaigns also seemed appealing with the inclusion of a choice of incentives like alcohol, money, and HIV tests as a key component of any demand-creation strategy. Some participants stated that they would be open to receiving services and information on HIV prevention services in the clinic if there is a designated area specifically for men.

## Discussion

In this study among predominantly heterosexual men 18–40 years in South Africa, we found that over 60% would be willing to use a mono-PrEP implant as an HIV prevention option, and for almost 20% of men, this would be their preferred prevention method. We found that men valued their health over cultural expectations and stigma and that they would disclose mono-PrEP implant use to their partners.

The monthly pill was the most preferred prevention option among men, followed by a six-monthly injectable and then an implant. This is supported by findings from a discrete choice experiment in Cape Town, South Africa where men also indicated a preference for an injectable over an implant [53]. Contrary to our findings, two online surveys with MSM in the United States, found that the implant was one of the most preferred HIV prevention methods of choice compared to oral PrEP and injection [35,54]. Heterosexual men may prefer oral PrEP, regardless of the dosing regimen, as it gives them a sense of control and active involvement in their prevention [55]. Furthermore, long-acting prevention methods such as implants and injectables are novel products, particularly among heterosexual men and they have concerns about the insertion process, side effects and removal procedures as found in the study. Currently, there is limited information about the safety and efficacy of the mono-PrEP implant, which may have influenced the participant’s choice. Preference for a two-monthly injectable was low, although it is notable that its introduction and evaluation in real-world settings among women and men in South Africa is imminent.

The most important prevention product considerations for men in this study were product side effects, the potential for long-term protection, and dual (e.g., STI and HIV) protection. This is consistent with findings from a study in New York: MSM were interested in long-acting products over daily oral PrEP, if it had high efficacy [56]. Long-acting products may alleviate pill burden and adherence issues for PrEP users, but additional issues like persistence with injections over time require further understanding [56,57]. Product efficacy did not significantly influence product choice in our study, although has been reported to have a strong influence among women in South Africa and Kenya [58], and doubts about PrEP efficacy have been reported as a barrier to uptake among young people in Sub-Saharan Africa [59].

Men were mainly concerned about the impact of side effects on their lives, particularly those affecting their sexual performance or fertility and the discomfort of implant insertion. A study of South African youth found that perceived side effects and concerns about physical activities could affect the uptake and use of a potential PrEP implant [60]. Ahead of any product introduction, clear and careful messaging is needed to dispel myths and prepare end-users for known side effects.

Disclosing product use in a relationship was important for men as they believed that their partner would be supportive and encourage clinic visits. Little is known about the relationship between PrEP disclosure and effective use among heterosexual men in South Africa but among AGYW, disclosing PrEP use elicited social support and influenced continuation and adherence [61]. The importance of individualised peer support that includes assistance with disclosure of PrEP use among MSM to people they deem important has also been noted [62].

The workshops revealed that men wanted to hear about the mono-PrEP implant on main media (radio and television) and social media platforms as well as through influencers and celebrities. Shamu et al (2021) found young men 18–24 years in South Africa preferred to receive information about PrEP on social media, school visits and TV adverts [63]. Social media is accessible, has been successful in delivering health information across various groups of people [64,65], and among Black and Latinx women and MSM in America has been found, in addition to custom mobile applications, to increase PrEP uptake, adherence, and awareness, [64].

The workshops also revealed that men preferred to learn about HIV prevention products in places that they frequented such as car washes, sports events, and taverns, as well as through community health campaigns. Men can be reached and encouraged to participate in HIV services through group meetings and community-based activities (such as sports, dancing competitions, debates, and other events) [66,67] which provide a safe environment to learn and share, receive social support, improve self-esteem and self-efficacy and decrease social isolation [67]. Incentives such as alcohol, money, and HIV tests were reported in our study to be important in drawing people to these campaigns. To ensure awareness and access to health information as well as encourage product use, programs, campaigns, and demand creation strategies should be tailored to the unique preferences of different users.

## Strengths and limitations

This study is one of the first studies in South Africa to describe the potential uptake of a mono-PrEP implant and assess the demand creation tactics preferred by men in South Africa. It contributes to the dearth of literature on heterosexual men and HIV prevention methods, particularly long-acting PrEP methods. It also contributes to the growing literature on perceptions and potential uptake of the mono-PrEP implant. Whilst this study was conducted in three provinces, representing a variety of geographical areas, we recognise that the results are not generalisable, due to the small sample size.

One data collection method was observation: observing participants engaging in workshops and sharing their views about existing and near-market HIV prevention products. Observations rely on the purposive selection of what information is important to note down [47]. Each observation already contains an element of interpretation of what is important to the observer and therefore introduces bias [47]. To minimise this bias, at least two project staff were observers, and the observation notes were consolidated to provide a holistic overview of the workshop discussions. Our findings are based on self-reported responses and participants may have underestimated their HIV and STI risk when reporting their perceived risk. Furthermore, given that our results are based on the potential uptake and use of prevention products that are not yet available in the country, preference for the mono-PrEP implant and demand creation tactics may differ once these methods become available. Actual uptake, once products are available, may differ from participants’ self-reported intentions and may be influenced by factors not able to be fully assessed through this study.

During recruitment, participants were pre-screened to assess eligibility for study participation. No biomedical assessment of HIV status was conducted; therefore, their response may reflect social-desirability bias and impact the accuracy of results. There may have been additional social desirability bias among participants who attended the workshop if they believed that facilitators wanted to hear positive feedback about the mono-PrEP implant. To mitigate this bias, the survey was developed to be self-completed, with de-identified data being collected. The study team also reassured participants that there were no ‘wrong’ or ‘right’ answers before completing the survey.

## Conclusion

Our study contributes to the small but growing field of mono-PrEP implants with several key findings that would influence and inform the development of novel HIV prevention methods. The most important product characteristics to men are side effects profile and dual and long-term protection. Further research into people’s knowledge and understanding of product efficacy would be an important next step in understanding product choice and subsequent uptake. Males in South Africa are interested in long-acting prevention products; however, they do have some concerns about the mono-PrEP implant. Therefore, they will require a comprehensive and participatory introduction to the implant to improve acceptability and address their concerns. This will ensure that existing gaps are filled, and that choice is expanded. Developers and implementers need to engage end-users in the development of HIV prevention products to identify their product-specific needs and implementation approaches that will cater to their lifestyles, as this will result in better uptake of the products. When an implant is available demand creation strategies using social media and health campaigns should be considered to engage and reach males.

## Supporting information

S1 Table Factors associated with choosing a mono-PrEP implant as a preferred prevention product among men (N = 142). (DOCX)
