# Supplementary material for: Factors influencing the uptake of a mono-PrEP implant for the prevention of HIV: Males’ perspectives from three South African provinces
Source: PLoS One. 2024 Jan 2;19(1):e0296341. doi: 10.1371/journal.pone.0296341 (PMC10760848; doi:10.1371/journal.pone.0296341)
Supplement: S1 Table — (DOCX) [file pone.0296341.s001.docx]

### **S1 Table 1**

### Factors associated with choosing a mono-PrEP implant as a preferred prevention product among males.

Adjusting for age and workshop attendance, participants residing in Gauteng were more likely to choose a mono-PrEP implant as a prevention product (OR 3.89 95% CI 1.08 – 14.69) compared to those residing in KwaZulu Natal. No other factors were associated with choice of a mono-PrEP implant compared to other prevention choices among men (Supplementary Table 1).

Supplementary Table 1: Factors associated with choosing a mono-PrEP implant as a preferred prevention product among men (N=142)

| **Variables** | **Controlled for age and workshop attendence** | |
| --- | --- | --- |
| **Study site** |  | **p value** |
| KZN | **Reference** |  |
| Gauteng | 3.89 (1.08 – 14.69) | **0.038** |
| Eastern Cape | 2.08 (0.49 - 8.80) | 0.321 |
| **Sexual orientation** |  |  |
| Heterosexual | **Reference** |  |
| Bisexual/Homosexual | 0.42 (0.05 – 3.54) | 0.427 |
| **Employment status** |  |  |
| Student | **Reference** |  |
| Employed (full/part time/self) | 0.34 (0.08 - 1.40) | 0.136 |
| Unemployed | 0.45 (0.13 - 1.49) | 0.191 |
| **Early sexual debut** |  |  |
| Yes | 0.70 (0.22 - 2.23) | 0.547 |
| **Relationship status** |  |  |
| Single | **Reference** |  |
| Casual partners | 0.45 (0.15 - 1.35) | 0.156 |
| Married or committed relationship | 1.01 (0.36 - 2.87) | 0.980 |
| **Knowledge of primary partner's HIV status** |  |  |
| Unknown | **Reference** |  |
| Known | 1.37 (0.59 - 3.20) | 0.470 |
| **More than one sexual partner** |  |  |
| No/Missing | **Reference** |  |
| Yes | 0.83 (0.34 – 2.03) | 0.679 |
| **Condom use at last sex** |  |  |
| No | **Reference** |  |
| Yes | 0.91 (0.40 - 2.09) | 0.826 |
| **Ever had transactional sex** |  |  |
| Yes | 1.39 (0.58 - 3.37) | 0.461 |
| **Ever tested for HIV** |  |  |
| Yes | 2.31 (0.27 – 19.80) | 0.444 |
| **Perceived risk of HIV** |  |  |
| At risk | 2.09 (0.86 - 5.07) | 0.102 |
| **Ever heard of PrEP** |  |  |
| Yes | 1.09 (0.44 -2.71) | 0.840 |
| **Ever used PrEP before** |  |  |
| Yes | 0.60 (0.20 - 1.76) | 0.350 |
| **Ever used PEP before** |  |  |
| Yes | 0.88 (0.27 - 2.91) | 0.838 |
| **Side effects are manageable** |  |  |
| Yes | 0.63 (0.23 - 1.74) | 0.382 |
| **Offers long term protection** |  |  |
| Yes | 1.44 (0.54 - 3.87) | 0.464 |
| **Provides dual protection** |  |  |
| Yes | 1.55 (0.50 – 4.86) | 0.452 |
